# Second harmonic generation microscopy provides accurate automated staging of liver fibrosis in patients with non-alcoholic fatty liver disease

**DOI:** 10.1371/journal.pone.0199166

**Published:** 2018-06-20

**Authors:** Pik Eu Chang, George Boon Bee Goh, Wei Qiang Leow, Liang Shen, Kiat Hon Lim, Chee Kiat Tan

**Affiliations:** 1 Department of Gastroenterology & Hepatology, Singapore General Hospital, Singapore; 2 Duke-NUS Medical School, Singapore; 3 Department of Anatomical Pathology, Singapore General Hospital, Singapore; 4 Biostatistics Unit, Yong Loo Lin School of Medicine, National University of Singapore, Singapore; University College London, UNITED KINGDOM

## Abstract

**Background:**

Assessment of severity of liver fibrosis is essential in the management of non-alcoholic fatty liver disease (NAFLD). Second Harmonic Generation (SHG) microscopy is a novel optical tissue imaging system that provides automated quantification of fibrosis based on unique architectural features of collagen. This study aims to develop and validate a SHG-based index for automated staging of liver fibrosis in patients with NAFLD.

**Methods:**

SHG microscopy was performed on archived liver biopsy specimens from 83 patients with NAFLD. A unique algorithm was developed to identify specific SHG parameters that correlated with fibrosis stage. The accuracy of the algorithm was compared against clinical assessment by experienced liver histopathologists using the Brunt fibrosis staging and further validated using the leave-one-out cross-validation method.

**Results:**

Mean age of the study cohort was 51.8 ± 11.7 years, with 41% males. A fibrosis index (SHG B-index) was developed comprising 14 unique SHG-based collagen parameters that correlated with severity of NAFLD fibrosis in a continuous fashion. The SHG B-index had excellent correlation with Brunt fibrosis stage (Spearman’s correlation 0.820, p<0.001). AUROCs for prediction of Brunt fibrosis stages 1, 2, 3 and 4 were 0.853, 0.967, 0.985 and 0.941 respectively. In the cross-validation analysis, the SHG B-index demonstrated high specificity for diagnosis of all grades of fibrosis. A SHG B-index score of >1.76 had an overall diagnostic accuracy of 98.5% for prediction of presence of bridging fibrosis (Brunt stage ≥3) with sensitivity of 87.5%, specificity 98.0%, positive predictive value 96.6% and negative predictive value 92.6%.

**Conclusion:**

The SHG B-index is a unique SHG-based index that provides accurate automated assessment of fibrosis stage in NAFLD patients.

## Introduction

Non-alcoholic fatty liver disease (NAFLD) is a spectrum of disorders characterized by excessive accumulation of hepatic fat associated with insulin resistance [1]. The prevalence of this disease is increasing worldwide in tandem with the obesity epidemic in developed countries. It is currently the most common liver condition in Western countries, affecting 17–46% of the adult population [2]. Worryingly, the prevalence of NAFLD exceeds 90% in patients with severe obesity and up to 69% in patients with type 2 diabetes mellitus [3]. NAFLD is histologically characterized into nonalcoholic fatty liver (NAFL) and nonalcoholic steatohepatitis (NASH) [4]. While NAFL is generally benign with low risk of progression of liver disease, NASH covers a wide spectrum of disease activity including fibrosis, cirrhosis and hepatocellular carcinoma (HCC) [1,2]. The severity of fibrosis is the most important prognostic factor in NAFLD and is the strongest predictor for the development of liver cirrhosis, need for liver transplantation, development of HCC and liver-related death [5,6]. Assessment of liver fibrosis is thus essential in the management of NAFLD.

The current gold standard for assessing liver fibrosis is with liver biopsy [7]. In the management of NAFLD, liver biopsy is essential both for the diagnosis of NASH and for fibrosis staging [8]. While serum biomarkers and transient elastography are acceptable non-invasive tests for screening of NAFLD patients with low risk of advanced fibrosis, reliable identification of at-risk patients with advanced fibrosis still requires confirmation by liver biopsy [2,9]. Unfortunately, liver biopsy is an imperfect gold standard due to sampling variability and inter-observer variability [10–13]. The NASH Clinical Research Network (NASH-CRN) system is the current reference histological scoring system used for assessment of fibrosis in NAFLD [14]. Severity of fibrosis is based on the distribution of fibrosis with initial peri-sinusoidal fibrosis (stage 1), progressing to portal/peri-portal fibrosis (stage 2), bridging fibrosis (stage 3) and finally cirrhosis (stage 4) [15]. Despite widespread adoption of this histological scoring system, poor inter-observer agreement remains a challenge [16]. Furthermore, the semi-quantitative nature of the fibrosis scoring system creates potential for error when the differentiation between groups is not distinct. Given that the process of fibrogenesis and fibrolysis is dynamic, an objective quantitative assessment on a continuous scale provides greater accuracy than a semi-quantitative score, especially when assessing changes in fibrosis over time [17,18]. This has important implications in the current era of antifibrotic therapy where linear changes in fibrosis quantification may be more meaningful to demonstrate response to therapy compared to the need to demonstrate stage migration.

There is thus an unmet need for a robust and accurate automated system of fibrosis assessment that will reduce inter-observer variability in the assessment of NASH fibrosis. In addition, this system should provide a continuous scale of fibrosis assessment that objectively evaluates changes in fibrosis severity. Most therapeutic trials for NASH require demonstration of histological improvement in fibrosis as a hard end-point to demonstrate efficacy. Such an automated fibrosis assessment tool would facilitate wide-scale international multi-center therapeutic trials by providing an efficient and reliable system for assessment of liver fibrosis.

Second harmonic generation (SHG) microscopy is a novel tissue imaging system based on non-linear optical microscopy which enables observation of endogenous tissue signals such as Two-Photon Excitation Fluorescence (TPEF) and Second Harmonic Generation (SHG) in unstained tissue samples [18]. TPEF provides visualization of the background liver architecture while the SHG signal provides accurate identification of fibrillar collagen [19,20]. SHG microscopy has been demonstrated to be reliable for fibrosis staging in chronic hepatitis B (CHB), outperforming collagen proportionate area measurement [21]. Computerized algorithms based on SHG pattern recognition and logic modelling allow automated determination of severity of fibrosis with the added advantage of objectivity, reproducibility and fast turnaround time [22]. However, the SHG fibrosis parameters in NAFLD are different from those in CHB due to differences in the distribution and nature of fibrosis. There is thus a need for NAFLD-specific SHG algorithms that accurately predict fibrosis stage based on Brunt rather than METAVIR fibrosis scoring. Proof-of-concept studies have provided encouraging evidence that support the role of SHG microscopy for evaluation of NASH fibrosis [23,24]. However, the reliability of SHG-based automated assessment for NAFLD fibrosis staging is not well established and requires validation.

We have developed an optical microscopy system that can scan and analyze the SHG properties of collagen in unstained liver tissue specimens. In this study, we aim to develop an algorithm based on SHG properties that are unique to NAFLD fibrosis and to validate its reliability to predict severity of fibrosis in patients with NAFLD.

## Methods

### Patient biopsy samples

Archived formalin-fixed, paraffin-embedded liver biopsy samples from 83 patients with NAFLD were retrieved for analysis. Subjects were identified from a database of patients in the Department of Gastroenterology and Hepatology of the Singapore General Hospital with a clinical diagnosis of NAFLD who underwent liver biopsy for clinical indications between 2005 and 2015. All subjects fulfilled the definition of NAFLD with >5% hepatic steatosis on liver biopsy in the absence of viral hepatitis, significant alcohol consumption (defined as >21 units of alcohol per week in males and >14 units in females), autoimmune and hereditary liver diseases [4]. Liver biopsy was performed for diagnostic and prognostic indications. The liver biopsy samples were processed as per routine clinical practice. The study protocol was approved by the SingHealth Centralized Institutional Review Board (CIRB Reference No: 2015/2527). 5μm-thick sections were cut from archived liver biopsy tissue and the anonymized specimens were sent for SHG imaging. The same sections were subsequently stained with hematoxylin and eosin (H&E) stain for blinded assessment by the histopathologists. Special histochemical stains such as trichrome and Victoria blue for assessment of liver fibrosis were available in the immediate preceding sections. Biopsy samples were reported as per clinical protocol by experienced liver histopathologists and severity of liver fibrosis was scored using Brunt’s classification [15].

### Image analysis

Unstained tissues of the 83 liver biopsy samples were imaged by the Genesis^™^ system (HistoIndex Pte. Ltd., Singapore). SHG microscopy was used to visualize collagen and two-photon excited fluorescence (TPEF) microscopy was utilized for visualization of the other cell structures. The settings of laser were similar to previous publications [22]. Images were acquired at 20× magnification with 512 × 512 pixels resolution, and each image tile had a dimension of 200 × 200 μm. A previous study had shown that SHG/TPEF technology-based quantification method was less sensitive to sampling error [22]. Thus, 10 five-by-five multi-tile images were randomly acquired for each sample with a final sampling size of 10 mm^2^ instead of scanning the entire biopsy tissue.

**Fig 1:**
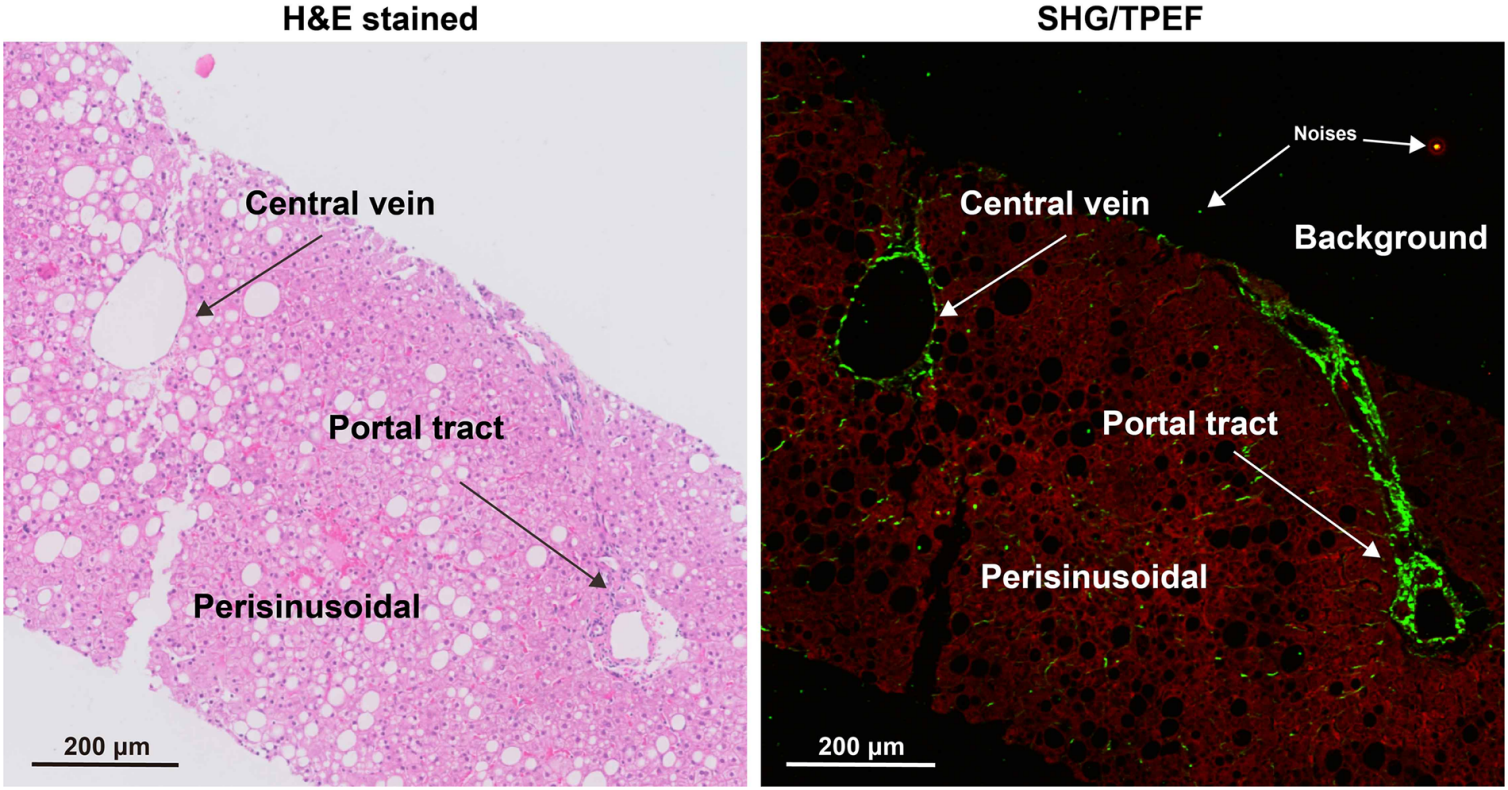
Illustration of central vein, portal tract and perisinusoidal regions in H&E stained image and SHG/TPEF image. H&E: hematoxylin & eosin, SHG: second harmonic generation, TPEF: two-photon excited fluorescence.

Landmark structures such as the portal tract and central vein were marked out on the H&E-stained slide and correlated to various variables on the SHG image (Fig 1). This allowed us to distinguish fibrosis occurring in 3 separate regions: central vein region (CV), portal tract region (PT) and peri-sinusoidal region (PS). An image processing algorithm was used to quantify fibrosis features in these three specific regions. Image analysis was divided into two parts: (1) determining specific regions, (2) quantifying collagen features in each region. The images were processed and analyzed using MATLAB 8.3 (The MathWork, USA).

**Fig 2:**
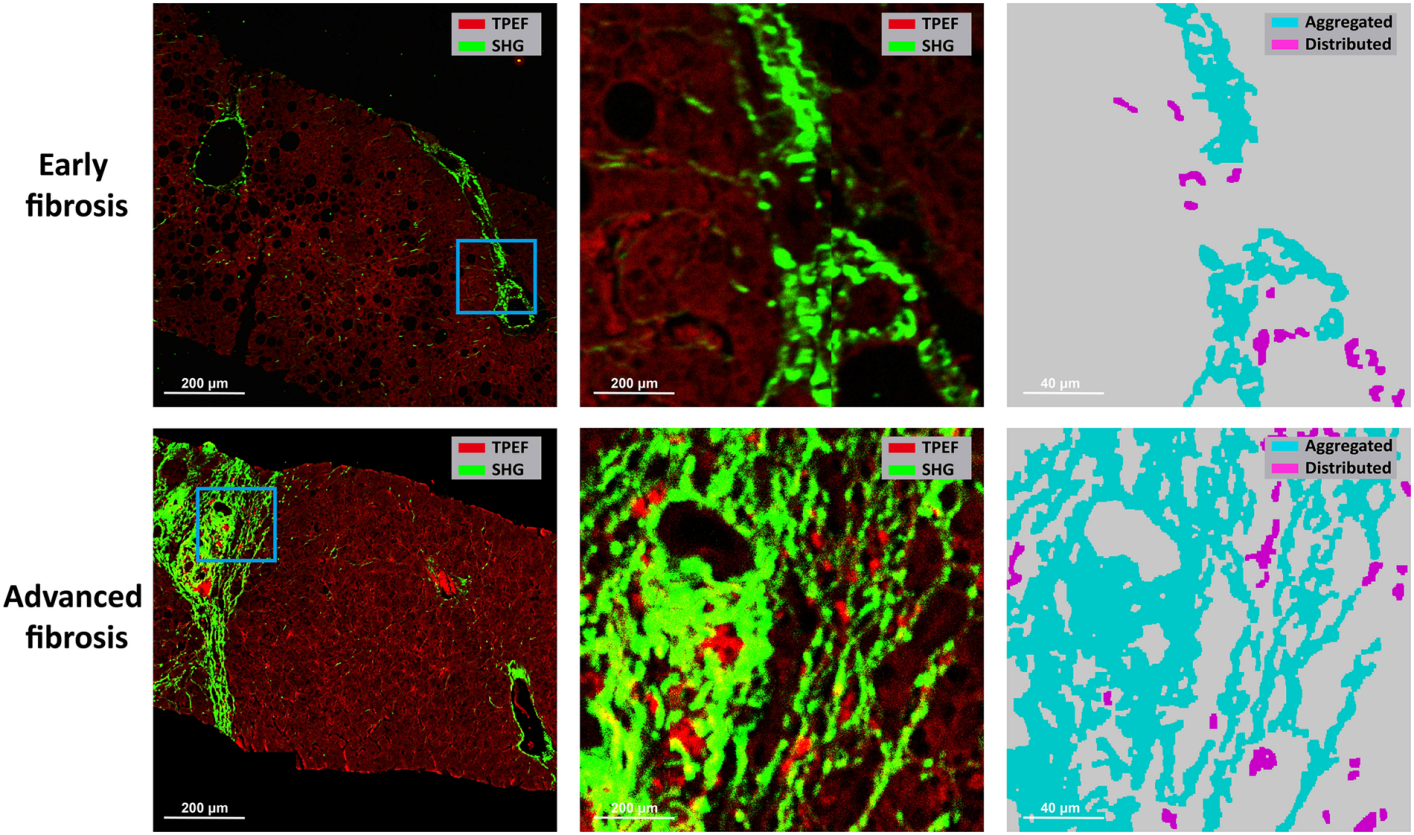
Illustration of specific collagen distribution at central vein, portal tract and perisinusoidal regions in early versus advanced fibrosis.

**Fig 3:**
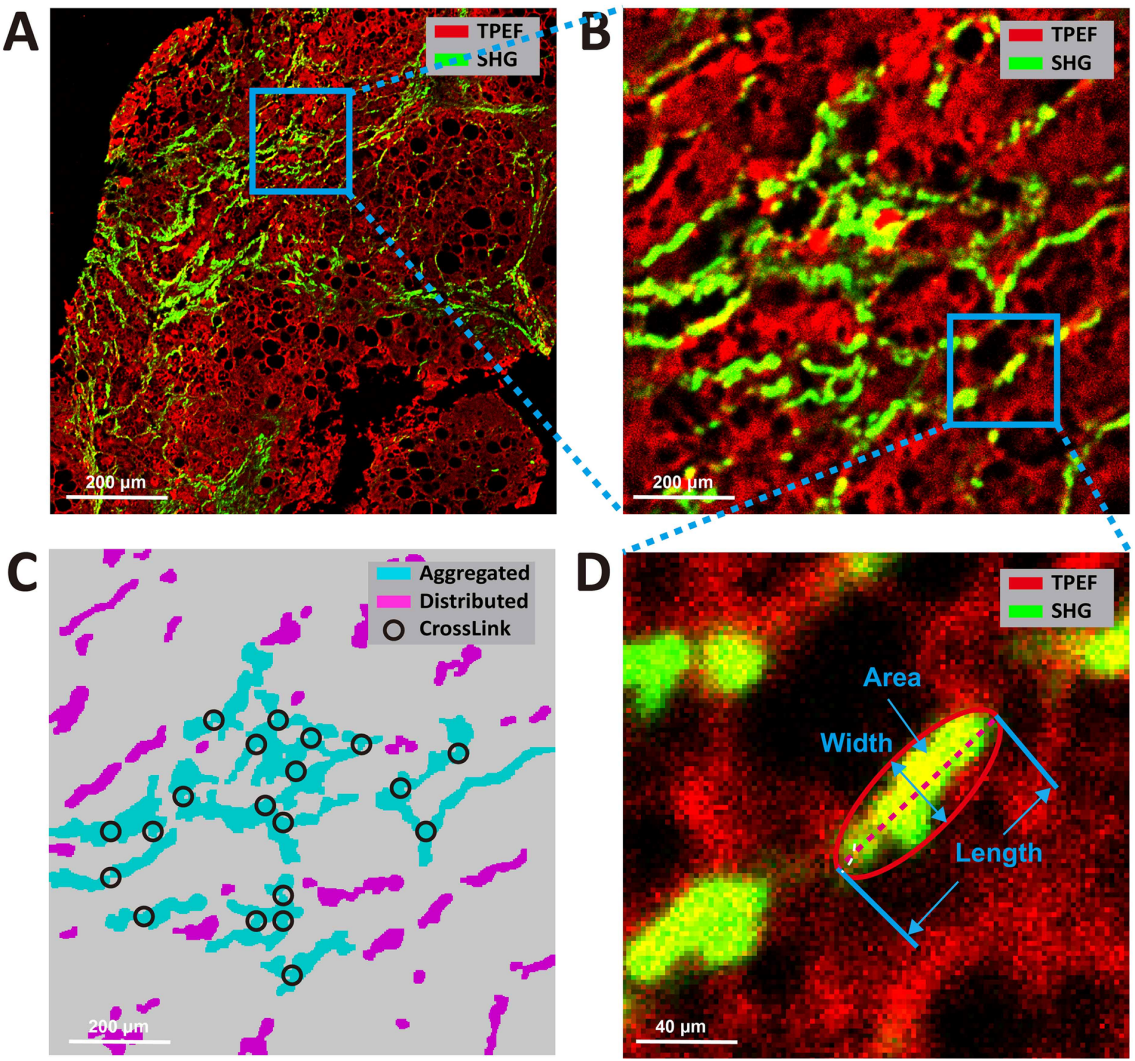
Illustration of collagen parameters on SHG microscopy. A) The RAW SHG/TPEF image. (B)The high magnification imaging performed in the area delimited by the blue square in picture A. (C) The aggregated and distributed strings of picture B. (D) The high magnification imaging performed in the area delimited by the blue square in picture B, and illustration of the width, length and area of a string. The thick string is by definition length-width ratio > 0.25.

1. ***Determining specific regions***: The SHG signal and TPEF signal in the background of SHG/TPEF images were recognized as noises, which were firstly removed (Fig 1). Collagen was detected from the SHG channel by the Ostu’s automatic threshold method [25]. The holes inside biopsy tissues which were caused by vessels, bile ducts, steatosis and unnatural cracks, were detected from the TPEF channel. Based on the features of each detected hole, such as density of hole, width and length of hole, hole solidity and the area of surrounding collagen of hole, the holes caused by vessels and bile ducts were distinguished from others by a decision tree, which was constructed by classification and regression tree (CART) method [26]. Multiple structures were then constructed based on the distances among the holes and the surrounding collagen of these holes. Each structure represented a portal tract region or a central vein region. Finally, these two regions were separated by another CART with features, such as the number of holes, the total area of holes, the area of maximal hole, and the area of collagen. The rest of the tissue area was regarded as perisinusoidal regions. This allowed the identification of specific collagen distribution at the central vein, portal tract and perisinusoidal areas in various stages of fibrosis (Fig 2).
2. ***Quantifying collagen features in each region***: In each region, aggregated collagen and distributed collagen were separated based on the cross-linking properties (Fig 3). Aggregated collagen was cross-linked, whereas distributed collagen was not. A total of 28 features including the percentages of different collagen patterns (all collagen, aggregated collagen and distributed collagen) and collagen string features (such as number of strings, numbers of short/long/thick/thin strings, numbers of short/long/thick/thin aggregated/ distributed strings, etc.) were extracted in each region. We used the relative thickness of collagen strings to each other to differentiate between thick and thin collagen strings. Thick strings were defined as collagen strings with a width/length ratio > 0.25. Another 16 similar features were extracted from the entire biopsy tissue. In total, 100 collagen features were extracted and quantified.

### Development of SHG algorithm for assessment of fibrosis

To automate the assessment of fibrosis stages of patients with NAFLD, a prediction model was developed based on the quantified collagen features. Firstly, feature selection was performed to reduce the dimensionality of data by selecting only a subset of quantified features. A common method of feature selection, named sequential feature selection was used in this study [27]. In the procedure of sequential feature selection, linear regression model was used whereby the criterion was residual sum of squares and the search algorithm was sequential forward selection. All the 83 subjects were employed to find the most significant collagen features related with Brunt stages. Fourteen features were finally selected, including the percentage of aggregated collagen (Agg), the width of strings (StrWidth), the percentage of central vein collagen (CV), the number of thick strings in CV (NoThickStrCV), the area of string in CV (StrAreaCV), the length of strings in CV (StrLengthCV), the length of aggregated strings in CV (StrLengthCVA), the area of distributed strings in CV (StrAreaCVD), the number of thin and aggregated strings in PT (NoThinStrPTA), the number of thick and distributed strings in PT (NoThickStrPTD), the number of distributed strings in PS (NoStrPSD), the number of short and distributed strings in PS (NoShortStrPSD), the area of distributed strings in PS (StrAreaPSD) and the length of distributed strings in PS (StrLengthSFD) (S1 Table).

Next, the training process was performed on all the 83 subjects and the validation process was performed using leave-one-out cross-validation method [28]. The selected features were used to assess the severity of liver fibrosis of NAFLD patients using a prediction model called the SHG B-index, which was constructed from all 83 subjects with multivariable linear regression method. If the result of the prediction model was less than 0, it was set to 0. Thus the severity of liver fibrosis was greater than 0 and may be greater than 4. To estimate how accurately the prediction model will perform in practice, leave-one-out cross-validation method was used (S1 Fig). Briefly, one sample is randomly retained as the validation data while the remaining 82 samples are used as training data to construct the prediction model. The performance of the prediction model is then tested on the single validation sample. The cross-validation process is repeated 83 times, with a different sample left out each time. The overall performance of the prediction model is assessed based on the sensitivity, specificity, positive and negative predictive values on the 83 predicted SHG B-indexes.

### Statistical analysis

Clinical and biochemical data were retrieved from the hospital’s computer database system. Statistical analysis was done with SPSS version 23 (Chicago, IL, USA). Quantitative fibrosis assessment by the SHG B-index was correlated with Brunt fibrosis score using Spearman correlation. AUROC analysis was performed to evaluate the accuracy of SHG B-index for prediction of the different grades of Brunt fibrosis. Optimal cut-off values of the quantitative SHG B-index were identified for prediction of Brunt fibrosis grades. Leave-one-out cross-validation (described above) was used to validate the performance of the SHG B-index model. The sensitivity, specificity, positive and negative likelihood ratios of SHG B-index for prediction for presence of bridging fibrosis (i.e. Brunt fibrosis grade 3) were calculated. Statistical significance level was set at p<0.05.

## Results

**Fig 4:**
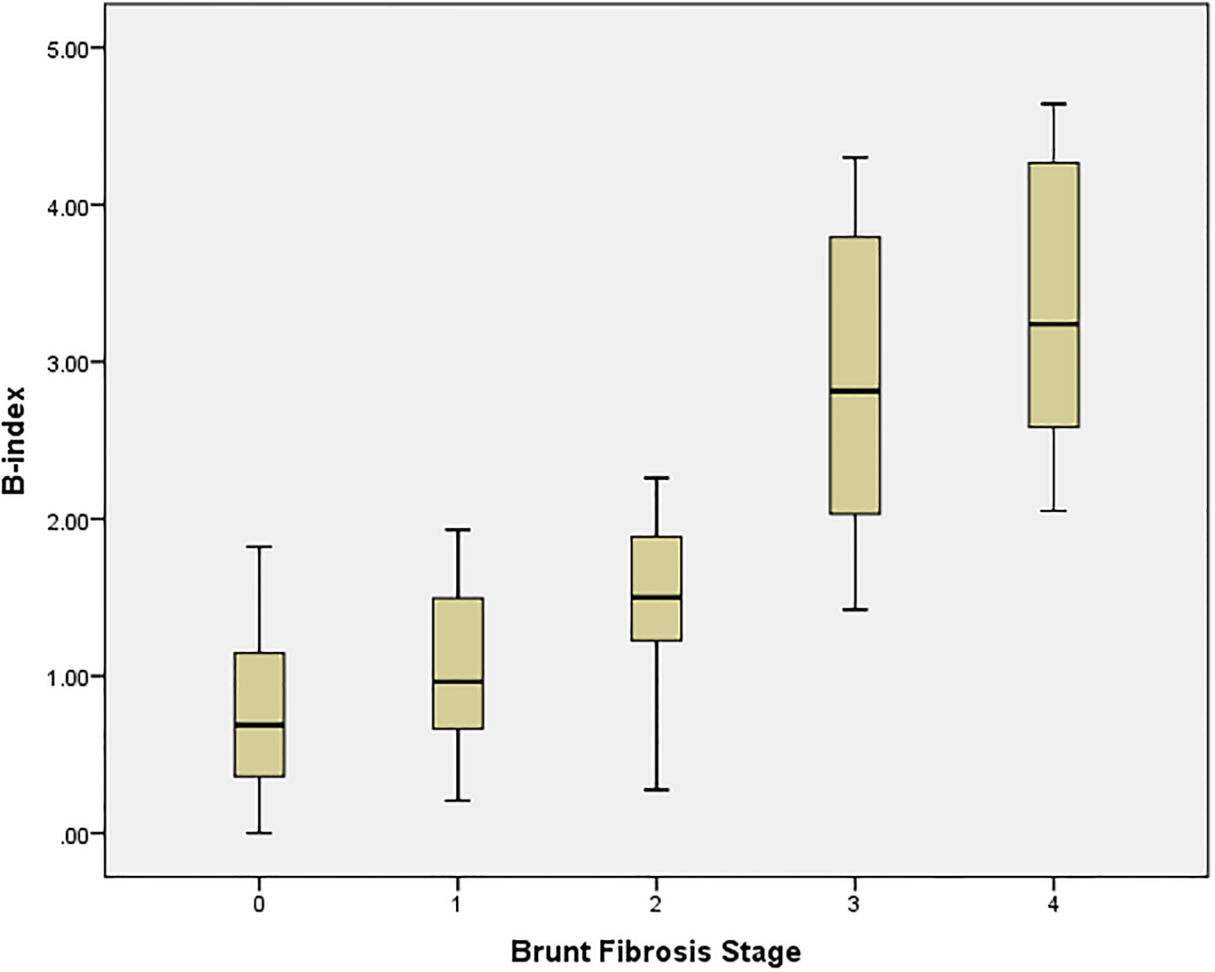
Boxplot of SHG B-index and Brunt fibrosis stage (n = 83).

Mean age of the study cohort was 51.8 ± 11.7 years, with 41% males and 77% of Chinese ethnicity. Demographic, anthropometric and biochemistry data are summarized in Table 1. Mean biopsy length, calculated by addition of all available fragments, was 2.4 ± 0.9 cm. Distribution of fibrosis in the study cohort was 22.9%, 30.1%, 8.4%, 16.9% and 21.7% for Brunt fibrosis stages B0, B1, B2, B3 and B4 respectively. Analysis of the SHG-derived data identified 14 SHG-based collagen parameters that correlated strongly with the Brunt classification. An algorithm was developed to provide a composite index of SHG-parameters that provided a quantitative assessment of fibrosis severity (S2 Table). This fibrosis index thus derived is termed the SHG B-index. The SHG B-index had a high correlation of 0.820 (p<0.001) with Brunt fibrosis staging, with an excellent ability to differentiate advanced fibrosis (Brunt stage 3 and 4) from no or mild fibrosis (Brunt stage 0 and 1). However, there was considerable overlap between Brunt stages 0, 1 and 2 (Fig 4).

**Table 1:** Baseline characteristics of NAFLD cohort.

	All subjects (n = 83)
Mean age (years)	51.8 ± 11.7
Male gender	34 (41%)
Chinese ethnicity	64 (77%)
BMI (kg/m^2^)	28.4 ± 6.9
HbA1c (%)	6.9 ± 1.2
Albumin (g/L)	42.7 ± 29.0
Bilirubin (μmol/L)	19.6 ± 26.4
Alanine transaminase (U/L)	80.9 ± 56.7
Aspartate transaminase (U/L)	62.3 ± 44.3
Platelet count (x10^9^/L)	220 ± 83
Prothrombin Time (s)	10.6 ± 1.3
Creatinine (mmol/L)	71.8 ± 24.7

**Fig 5:**
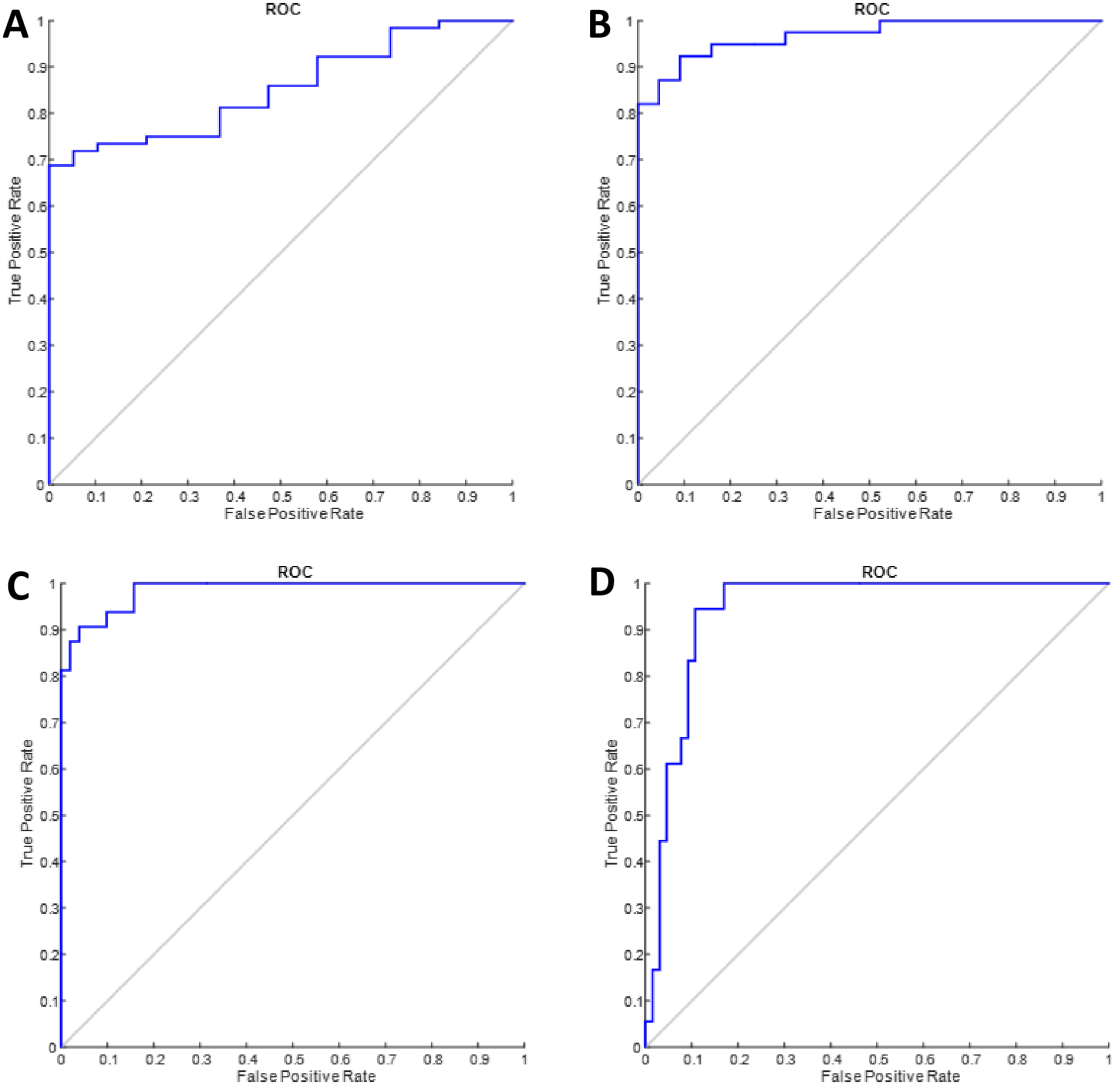
AUROCs of SHG B-index to predict fibrosis stages.

AUROC analysis was performed to evaluate the accuracy of the SHG B-index to identify the various fibrosis stages (Table 2). AUROC of SHG B-index to predict mild fibrosis was 0.853 (95% confidence interval 0.774–0.993), significant fibrosis 0.967 (95% confidence interval 0.933–1.000), bridging fibrosis 0.985 (95% confidence interval 0.966–1.000) and cirrhosis 0.941 (95% confidence interval 0.892–0.990) (Fig 5).

**Table 2:** AUROCs of SHG B-index for prediction of Brunt fibrosis.

NAFLD Fibrosis Group	Brunt fibrosis stage	AUROC	*P*	95% CI	SHG B-index cut-off value
Mild fibrosis	0 vs 1/2/3/4	0.853	<0.001	0.774–0.933	1.18
Significant fibrosis	0/1 vs 2/3/4	0.967	<0.001	0.933–1.000	1.33
Bridging fibrosis	0/1/2 vs 3/4	0.985	<0.001	0.966–1.000	1.76
Cirrhosis	0/1/2/3 vs 4	0.941	<0.001	0.892–0.990	2.76

Optimal cut-off values of SHG B-index to identify specific Brunt fibrosis groups were calculated using Youden’s index (maximal sum of sensitivity and specificity). To assess for presence of fibrosis (i.e. to differentiate between no fibrosis and presence of fibrosis), the optimal cut-off value of the SHG B-index was 1.18. The cut-off values for presence of significant fibrosis, advanced fibrosis and cirrhosis were 1.33, 1.76 and 2.76, respectively.

The performance of the SHG B-index was validated using the leave-one-out cross-validation method (Table 3). Overall, the SHG B-index performed very well with high specificity ranging from 84.2% to 98.0% for diagnosis of all grades of fibrosis. The SHG B-index was less sensitive for identification of mild fibrosis but it had a high positive predictive value with a value above 1.18 providing a 93.2% probability of presence of fibrosis. Conversely a SHG B-index value below 2.76 provided a 93.6% probability of not having cirrhosis. The SHG B-index performed best for the prediction of advanced fibrosis, which is particularly relevant in the management of NAFLD. A SHG B-index value greater than 1.76 had an overall diagnostic accuracy of 98.5% for prediction of presence of bridging fibrosis (Brunt stage ≥3) with sensitivity of 87.5%, specificity 98.0%, positive likelihood ratio 43.75 and negative likelihood ratio of 0.13, positive predictive value of 96.6% and negative predictive value of 92.6%.

**Table 3:** Cross-validation of SHG B-index to predict Brunt fibrosis stage.

NAFLD Fibrosis Group	Brunt fibrosis stage	SHG B-index cut-off	Sens	Spec	+LR	-LR	PPV	NPV
Mild fibrosis	0 vs 1/2/3/4	1.18	65.6%	84.2%	4.15	0.41	93.2%	41.0%
Significant fibrosis	0/1 vs 2/3/4	1.33	84.6%	86.4%	6.22	0.18	86.8%	86.7%
Bridging fibrosis	0/1/2 vs 3/4	1.76	87.5%	98.0%	43.75	0.13	96.6%	92.6%
Cirrhosis	0/1/2/3 vs 4	2.76	83.8%	89.2%	7.71	0.19	66.7%	93.6%

Sens = Sensitivity, Spec = Specificity

+LR = Positive likelihood ratio, -LR = Negative likelihood ratio

PPV = Positive Predictive Value, NPV = Negative Predictive Value

## Discussion

Using the unique capabilities of SHG to identify collagen parameters associated with NAFLD fibrosis, we developed a composite index (the SHG B-index) that has high accuracy for identifying various stages of NAFLD fibrosis. Using a cross-validation method, we confirm that the SHG B-index is reliable for the prediction of severity of liver fibrosis compared to traditional histopathologists’ assessment using Brunt classification. A SHG B-index of >1.76 is highly sensitive and highly specific for presence of bridging fibrosis, a clinically important feature that is associated with poor prognosis in NAFLD patients. This study thus provides evidence to support the potential clinical application of an automated system of fibrosis staging using unstained liver biopsy specimens.

The development of SHG-based optical microscopy represents a potential paradigm shift in the histopathological assessment of fibrosis, providing several advantages over conventional histopathological assessment. The unique crystalline triple-helix structure of fibrillar collagen provides a specific SHG signal that allows accurate identification, characterization and quantification of liver fibrosis [19]. This process does not require staining of tissues, thus reducing degradation of the sample and production of artifacts which impair the quality of the interpretation [21]. The ability to characterize fibrillar collagen within a 3-dimensional lattice framework provides the opportunity to evaluate the process of fibrosis remodeling, which is a unique advantage over the 2-dimensional assessment using standard histopathology [18]. Once the system has been calibrated, the method is highly reproducible, allowing standardization of measurement and reduction of interobserver variability. SHG-based microscopy thus has the potential to provide an automated assessment of fibrosis stage within hours of placing an unstained liver biopsy slide into a compact machine. From a clinical perspective, it is crucial to develop SHG-based algorithms that provide reliable assessment of fibrosis based on the specific etiology of disease, since current fibrosis staging systems tend to be disease-specific.

SHG-based assessment of liver fibrosis has been shown to be reliable in chronic viral hepatitis. Correlation between SHG signal and METAVIR fibrosis score in patients with chronic viral hepatitis was initially described by Gailhouste and colleagues [21] and subsequently validated in an independent study [22]. However, the distribution of fibrosis in NAFLD is different from that in viral hepatitis. In the former, fibrosis begins in the peri-cellular and peri-sinusoidal areas whereas fibrosis begins in the periportal regions in the latter. The SHG algorithms that were developed based on METAVIR staging thus cannot be applied to NAFLD. There is thus an important need to develop and validate SHG-based algorithms that are unique for NAFLD, paying particular attention to the ability to geographically differentiate fibrosis deposition in the central vein, portal tract and peri-sinusoidal areas. This is particularly relevant because most clinical liver biopsies are currently performed for NAFLD. As a testament to the developing interest in this area, a proof-of-concept study recently explored the use of SHG microscopy for assessment of fibrosis stage in NAFLD [24]. The authors identified four SHG-based parameters that reliably differentiated the various fibrosis stages with AUROCs of 0.81 to 0.92. However, their algorithm lacked the ability to differentiate portal vein fibrosis from central vein fibrosis. The results of our study thus validate the reliability of SHG as an automated method for fibrosis staging. In addition, our algorithm can localize and quantify central vein fibrosis, portal tract fibrosis and peri-sinusoidal fibrosis. This provides the potential to differentiate fibrosis from different etiologies, for example in patients with concomitant NASH and chronic viral hepatitis. Furthermore, the SHG B-index that we have developed provides a convenient single composite index that can be easily applied in routine clinical practice to provide automated assessment of fibrosis.

We observed that the quantitative distribution of fibrosis assessed by SHG B-index was highly sensitive and specific for the identification of advanced fibrosis in NAFLD but was less discerning in discriminating between early stages of fibrosis. In the early stages of fibrosis (Brunt stage 1 and 2), the amount of collagen in the liver is similar, but the distribution of collagen is different being predominantly peri-sinusoidal in stage 1 and peri-portal in stage 2. However, in advanced fibrosis (Brunt stage 3) we observe a significant increase in the SHG B-index, reflecting an increase in the amount of collagen deposited as the fibrous tissue progresses from the portal tracts to the central veins. The SHG B-index thus provides a reliable method to identify NAFLD patients with advanced fibrosis who are at increased risk of liver-related morbidity and mortality.

SHG microscopy has several important clinical implications in the management of NAFLD patients. Firstly, it provides a highly reliable automated assessment of fibrosis that can reduce interobserver variability and improve accuracy of histological staging of liver fibrosis. Secondly, it can be performed on unstained tissues thus avoiding the need for cumbersome staining procedures and degradation artifacts. Thirdly, SHG optical microscopy provides a quantitative assessment of fibrosis on a continuous scale which reflects dynamic change in fibrosis more accurately than the conventional semi-quantitative histological grading systems. With the increase in new anti-fibrotic therapeutic compounds, the use of a quantitative method with a continuous scoring scale to assess progression or regression in severity of fibrosis is intuitively more reflective of the true therapeutic response. The primary utility of SHG-based assessment of liver fibrosis may be in therapeutic NAFLD trials, many of which require serial liver biopsies for assessment of improvement in fibrosis. The automated fibrosis scoring provided by SHG microscopy helps to minimize inter-observer variability and reduces the cost and logistics associated with shipping of samples for central histology review. This automated, standardized and clinically reliable fibrosis assessment system may thus pave the way for future large, multicenter, international collaborative studies.

Although SHG optical microscopy offers several novel applications in fibrosis assessment, it is important to emphasize that this technology does not replace the need for invasive liver biopsy and cannot replace the key role of the histopathologist in clinical practice [18]. The information provided by the SHG B-index is only limited to fibrosis assessment whereas the histopathologists provides essential details on other features such as inflammation, steatosis, ballooning degeneration, Mallory-Denk bodies, iron deposition and exclusion of other liver diseases. It also does not overcome the problem of sampling error which is an inherent limitation of liver biopsy. For this reason, we feel that SHG-based fibrosis assessment is most suitable as an adjunct tool that provides additional value to the efficiency and reliability of the histopathologist in clinical practice. The SHG B-index can be used to provide an external reference to guide the histopathologist, thus minimizing inter-observer variability in assessment of fibrosis in NAFLD. This can help to reduce the clinical variability in fibrosis assessment using the NASH-CRN criteria among community-based histopathologists, thus improving the accuracy of fibrosis assessment in general clinical practice [16]. It can also be a useful training tool for less-experienced histopathologists by providing a guide for accuracy and standardization of fibrosis scoring.

We acknowledge that our study is limited by a modest sample size from a single center and lack independent external validation. Nonetheless, we have employed a robust methodology and analysis to provide reliable results, which we believe are important to the scientific community. A larger multi-center is currently being planned to validate the SHG B-index in an independent cohort. Another limitation is that this study did not examine changes in fibrosis stage from subjects with repeat liver biopsies. Hence, the role of SHG B-index for assessment of progression or regression of NAFLD fibrosis in longitudinal pre-and post-treatment liver biopsies needs to be validated as well, particularly in the context of response to pharmacological intervention for NAFLD.

In conclusion, this study demonstrates that the SHG B-index, a unique fibrosis index based on SHG optical microscopy, provides highly reliable automated assessment of fibrosis in patients with NAFLD. This index has a high specificity for advanced fibrosis which is a clinically important sentinel point in the management of NAFLD.

## Supporting information

S1 Table List of SHG parameters. (DOCX)

S2 Table Descriptive statistics of B-index prediction model. (DOCX)

S1 Fig Supplementary figure: Illustration of leave-one-out cross-validation. One sample is randomly retained as the validation set while the remaining 82 samples are used as the training set to develop a prediction model. The performance of the prediction model is then tested on the single validation sample. The cross-validation process is repeated 83 times, with a different sample left out each time. The overall performance of the prediction model to predict the various fibrosis stages is assessed based on the 83 predicted B-indexes.
(PDF)
