# Supplementary material for: Second harmonic generation microscopy provides accurate automated staging of liver fibrosis in patients with non-alcoholic fatty liver disease
Source: PLoS One. 2018 Jun 20;13(6):e0199166. doi: 10.1371/journal.pone.0199166 (PMC6010245; doi:10.1371/journal.pone.0199166)
Supplement: S1 Table — (DOCX) [file pone.0199166.s001.docx]

| **No.** | **Abbreviation** | **Description** | **Unit** |
| --- | --- | --- | --- |
| 1 | Agg | The percentage of aggregated collagen | % |
| 2 | StrWidth | The width of strings | μm |
| 3 | CV | The percentage of collagen in CV | % |
| 4 | NoThickStrCV | The number of thick strings in CV | n |
| 5 | StrAreaCV | The area of string in CV | μm^2^ |
| 6 | StrLengthCV | The length of strings in CV | μm |
| 7 | StrLengthCVA | The length of aggregated strings in CV | μm |
| 8 | StrAreaCVD | The area of distributed strings in CV | μm |
| 9 | NoThinStrPTA | The number of thin and aggregated strings in PT | n |
| 10 | NoThickStrPTD | The number of thick and distributed strings in PT | n |
| 11 | NoStrPSD | The number of distributed strings in PS | n |
| 12 | NoShortStrPSD | The number of short and distributed strings in PS | n |
| 13 | StrAreaPSD | The area of distributed strings in PS | μm^2^ |
| 14 | StrLengthSFD | The length of distributed strings in PS | μm |
