# Supplementary material for: Second harmonic generation microscopy provides accurate automated staging of liver fibrosis in patients with non-alcoholic fatty liver disease
Source: PLoS One. 2018 Jun 20;13(6):e0199166. doi: 10.1371/journal.pone.0199166 (PMC6010245; doi:10.1371/journal.pone.0199166)
Supplement: S2 Table — (DOCX) [file pone.0199166.s002.docx]

| **Descriptive Statistics** | | | | | | | | |
| --- | --- | --- | --- | --- | --- | --- | --- | --- |
| **Brunt** | **N** | **Range** | **Minimum** | **Maximum** | **Mean** | **Std. Error** | **Std. Deviation** | **Variance** |
| 0 | 19 | 1.15 | .00 | 1.15 | .7509 | .07912 | .34488 | .119 |
| 1 | 25 | 1.37 | .34 | 1.70 | .9650 | .07348 | .36740 | .135 |
| 2 | 7 | 1.61 | .86 | 2.48 | 1.5231 | .20624 | .54565 | .298 |
| 3 | 14 | 3.10 | 1.33 | 4.43 | 2.7854 | .27282 | 1.02081 | 1.042 |
| 4 | 18 | 3.02 | 2.32 | 5.34 | 3.6126 | .16225 | .68838 | .474 |
