# Supplementary figures and images for: Second harmonic generation microscopy provides accurate automated staging of liver fibrosis in patients with non-alcoholic fatty liver disease
Source: PLoS One. 2018 Jun 20;13(6):e0199166. doi: 10.1371/journal.pone.0199166 (PMC6010245; doi:10.1371/journal.pone.0199166)

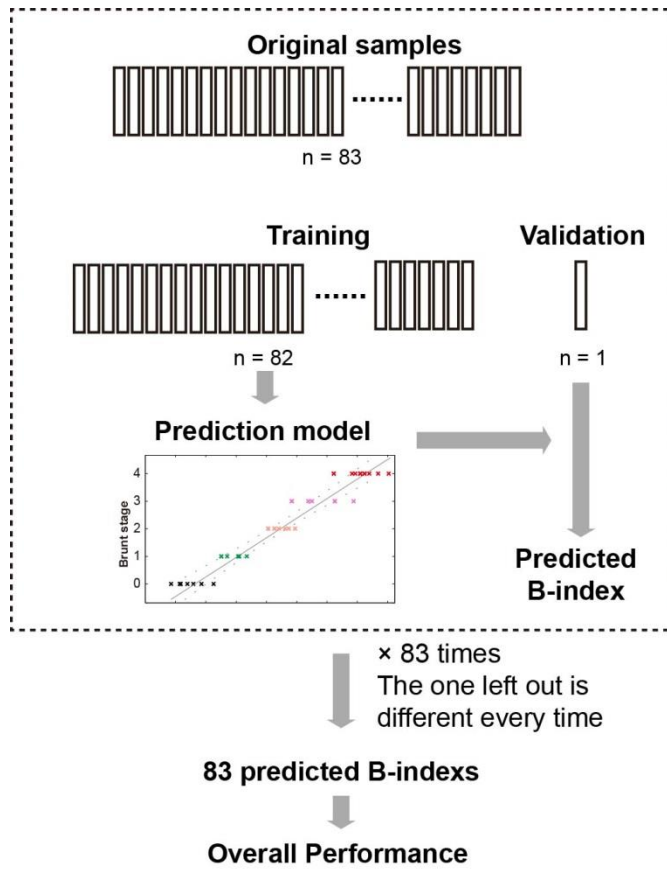

Supplement: S1 Fig — One sample is randomly retained as the validation set while the remaining 82 samples are used as the training set to develop a prediction model. The performance of the prediction model is then tested on the single validation sample. The cross-validation process is repeated 83 times, with a different sample left out each time. The overall performance of the prediction model to predict the various fibrosis stages is assessed based on the 83 predicted B-indexes. (PDF) [file pone.0199166.s003.pdf]
